# The (Null) Effect of Affective Touch on Betrayal Aversion, Altruism, and Risk Taking

**DOI:** 10.3389/fnbeh.2017.00251

**Published:** 2017-12-19

**Authors:** Lina Koppel, David Andersson, India Morrison, Daniel Västfjäll, Gustav Tinghög

**Affiliations:** ^1^Center for Social and Affective Neuroscience, Department of Clinical and Experimental Medicine, Linköping University, Linköping, Sweden; ^2^JEDI Lab, Division of Economics, Department of Management and Engineering, Linköping University, Linköping, Sweden; ^3^Division of Psychology, Department of Behavioral Sciences and Learning, Linköping University, Linköping, Sweden; ^4^Decision Research, Eugene, OR, United States; ^5^The National Center for Priority Setting in Health Care, Department of Medical and Health Sciences, Linköping University, Linköping, Sweden

**Keywords:** touch, oxytocin, betrayal aversion, altruism, risk taking, trust

## Abstract

Pleasant touch is thought to increase the release of oxytocin. Oxytocin, in turn, has been extensively studied with regards to its effects on trust and prosocial behavior, but results remain inconsistent. The purpose of this study was to investigate the effect of touch on economic decision making. Participants (*n* = 120) were stroked on their left arm using a soft brush (touch condition) or not at all (control condition; varied within subjects), while they performed a series of decision tasks assessing betrayal aversion (the Betrayal Aversion Elicitation Task), altruism (donating money to a charitable organization), and risk taking (the Balloon Analog Risk Task). We found no significant effect of touch on any of the outcome measures, neither within nor between subjects. Furthermore, effects were not moderated by gender or attachment. However, attachment avoidance had a significant effect on altruism in that those who were high in avoidance donated less money. Our findings contribute to the understanding of affective touch—and, by extension, oxytocin—in social behavior, and decision making by showing that touch does not directly influence performance in tasks involving risk and prosocial decisions. Specifically, our work casts further doubt on the validity of oxytocin research in humans.

## Introduction

Touch plays a vital role for social and psychological well-being and is said to have a “Midas effect” on judgments and decisions, promoting prosocial behavior (Crusco and Wetzel, 1984; Schirmer et al., 2016). Pleasant touch is also thought to increase the release of oxytocin (Walker et al., 2017). Oxytocin, in turn, has been extensively studied with regards to its effects on trust and prosocial behavior, but results remain inconsistent. In this study, we indirectly investigated the presumed effect of endogenously released oxytocin by gently stroking participants on their forearm while they performed a series of decision tasks assessing betrayal aversion, altruism, and risk taking. Our findings contribute to the understanding of touch—and, by extension, oxytocin—in social behavior and decision making.

The first evidence for a causal link between oxytocin and trust was provided by Kosfeld et al. (2005), who found that intranasally administered oxytocin increased investments in a trust game. However, this finding has been difficult to replicate. Some researchers have found that intranasal oxytocin has no effect on initial investments in the trust game, but that it influences investments following trust betrayal (Baumgartner et al., 2008); others have found that the effect of oxytocin on trust and responses to trust betrayal is moderated by gender (Yao et al., 2014) or that it only applies to individuals high in attachment avoidance (De Dreu, 2012). A recent review and meta-analysis found no consistent effect of intranasal oxytocin on trust (Nave et al., 2015). One potential caveat of these studies is the controversial assumption that intranasal oxytocin passes the blood–brain barrier and reaches target brain areas (Leng and Ludwig, 2016). However, studies correlating plasma levels of endogenously released oxytocin with trust have also yielded mixed results. Some researchers have found that oxytocin has no effect on investments in the trust game, but that it influences the amount returned by trustees (Morhenn et al., 2008) or that the level of oxytocin is higher following the receipt of an intentional monetary transfer compared to an equivalent transfer that is determined by a random lottery (Zak et al., 2005). Others have found a U-shaped pattern such that individuals who are either high or low in plasma oxytocin are both more trusting and more trustworthy than participants with moderate levels of oxytocin (Zhong et al., 2012). A drawback of several of these studies that could help explain the inconsistent findings is that they have used unextracted samples of plasma oxytocin that have been shown to be unreliable (McCullough et al., 2013; Christensen et al., 2014). In addition, the oxytocin literature suffers from issues such as publication bias (Lane et al., 2016) and low statistical power (Walum et al., 2016). In sum, the evidence that oxytocin directly influences behavior remains sparse. If there is an effect, it is likely moderated by a variety of factors.

In the present study, we aimed to experimentally manipulate the levels of endogenously released oxytocin by gently stroking participants' forearm with a soft brush. Slow, gentle touch is perceived as pleasant and activates areas of the brain that are associated with interoception and reward, such as the insula, caudate, and dorsolateral prefrontal cortex (Perini et al., 2015). Gentle stroking of the skin at a speed of 1–10 cm/s also activates a specific type of nerve fibers, C-tactile (CT) afferents, that respond optimally to the type of touch that is perceived as most pleasant (Löken et al., 2009). The pleasant and relaxing effects of CT-optimal touch mirror those of exogenously administered or endogenously released oxytocin, suggesting that activation of CT fibers increases the release of oxytocin (Walker et al., 2017, see also Uvnäs-Moberg et al., 2015), although this link has yet to be established empirically. Furthermore, it has been shown that people spontaneously stroke other humans, but not objects, at CT-optimal speeds (Croy et al., 2016), which supports the idea that touch, in particular the kind of touch that activates CT fibers, plays a vital role in the formation and maintenance of social bonds (Olausson et al., 2010). Thus, it seems reasonable to hypothesize that affective touch influences economic behavior; however, to the best of our knowledge, no such studies exist.

We investigate the effect of touch on betrayal aversion, altruism, and risk taking. Betrayal aversion refers to the reluctance to take risk when the outcome depends on a human counterpart rather than when it is determined by nature (i.e., chance; Bohnet and Zeckhauser, 2004). The first evidence for this tendency was provided by Bohnet and Zeckhauser (2004), who elicited participants' minimum acceptable probability (MAP) of getting an even split (the good outcome) for which they were willing to take a risk in a standard trust game compared to an equivalent risk-only trust game. They found that participants' MAPs were greater in the trust game than in the risk-only trust game, indicating that people infer a cost from the possibility of being betrayed by another person, above and beyond the monetary cost. This finding has been replicated across several cultures (Bohnet et al., 2008). More recent neuroimaging studies have shown that playing a trust game with a human counterpart rather than a computer activates areas of the brain that are associated with emotion regulation and negative affect, including the right anterior insula, medial frontal cortex, and right dorsolateral prefrontal cortex (Aimone et al., 2014). Furthermore, betrayal averse participants show less amygdala activity before choosing a risky compared to certain option in a non-social risk task but not in an equivalent social risk task and show greater activity in the striatum, which is involved in reward, after receiving a social than a non-social outcome (Lauharatanahirun et al., 2012).

Betrayal aversion has been suggested as one of the mechanisms by which oxytocin increases trust (Engelmann and Fehr, 2017). The prediction that follows is that touch, because it presumably increases oxytocin levels, reduces betrayal aversion. For instance, Baumgartner et al. (2008) gave male participants intranasal oxytocin or placebo and compared decisions made by investors in a trust game both before and after they received feedback that the trustees did not reciprocate in 50% of cases. Oxytocin had no significant effect on investments before feedback, but after feedback participants who had received oxytocin invested more than those who had received placebo. This suggests that oxytocin reduces the sensitivity to betrayal of trust. However, note that this study relied on intranasal oxytocin despite controversial underlying assumptions. More recent research has failed to replicate the findings (Klackl et al., 2013). Another study that is of particular relevance to the present study was conducted by Morhenn et al. (2008), who compared participants' behavior in a one-shot trust game following either a 15-min massage or a 15-min rest. They found no difference in investors' behavior, but trustees who had received a massage returned more money than trustees who had rested. Most importantly, for participants who had rested, both oxytocin levels and the amount received from the investor predicted the amount returned by the trustees, but for participants who had received a massage, only oxytocin predicted the amount returned. These findings suggest that touch—and oxytocin—promotes prosocial behaviors, although note that these researchers used unextracted samples of plasma oxytocin that may be unreliable (see McCullough et al., 2013; Christensen et al., 2014).

The effect of touch on altruism is more difficult to predict. Previous research suggests that touch increases positive valuations and makes people more prosocial overall, an effect known as the Midas effect (Crusco and Wetzel, 1984; Schirmer et al., 2016). For instance, restaurant guests give larger tips after having been touched on the shoulder by the waitress (Crusco and Wetzel, 1984) and people who have been touched are more likely to help a stranger (Kleinke, 1977; Guéguen and Fischer-lokou, 2003). The prediction that follows from this line of research is that touch increases altruism. However, the oxytocin literature gives a more complex picture. Some researchers have found that oxytocin increases donations to charitable organizations (Barraza et al., 2011; Marsh et al., 2015) and that it increases monetary contributions in a social dilemma (Israel et al., 2012). Others have found that the effect of oxytocin on altruism depends on contextual factors, such as whether the target is a stranger or a close other (Pornpattananangkul et al., 2017) and whether they belong to the in-group or to the outgroup (De Dreu et al., 2010). An alternative explanation of these inconsistencies is that oxytocin promotes mentalizing, i.e., the ability to take someone else's perspective (Domes et al., 2007; but see also Radke and de Bruijn, 2015, and Leppanen et al., 2017, who found no support of this suggestion). Zak et al. (2007) found that intranasal oxytocin increased monetary offers in an ultimatum game but not in an equivalent dictator game. The difference between these two tasks is that in the ultimatum game, the investor has to take the recipient's reaction into account because the recipient can reject the investor's offer, resulting in zero earnings for both players. In contrast, in the dictator game, the recipient simply obtains whatever amount the investor offers, which does not require perspective taking to the same extent. Furthermore, a recent fMRI study showed that oxytocin had no effect on the frequency of altruistic decisions, but that it increased activity in the left temporo-parietal junction, a region that has been implicated in theory of mind, when participants observed others being helped (Hu et al., 2016). Following this line of research, touch should have no direct effect on altruism. However, note again that these studies used intranasal oxytocin.

Oxytocin has mostly been studied in terms of its role in social relationships and behavior, so its effect on risk taking in the non-social domain is unclear. Physical contact has been shown to increase financial risk taking, especially if the toucher is female and if the touch involves a tap on the shoulder rather than a handshake (Levav and Argo, 2010). Somatosensory stimulation in the form of thermal pain also increases risk seeking (Koppel et al., 2017). On the other hand, individuals who have received oxytocin are not more risk seeking than participants who have received a placebo, as shown in studies comparing the effect of intranasal oxytocin on decisions in a trust game to decisions in an equivalent risk game (e.g., Kosfeld et al., 2005). To our knowledge, only one published study has investigated the effect of intranasal oxytocin using a risk-taking task that does not involve another person, and it found no main effect of oxytocin on risk taking (Patel et al., 2015). However, a three-way interaction appeared such that men (but not women) who had received oxytocin were less risk taking if they were told that others were watching them perform the task (which resulted in social stress). Thus, if touch influences risk taking, it may do so via some mechanism other than increased oxytocin, such as increased positive affect.

To the best of our knowledge, our study is the first to investigate the effect of CT-optimal touch on economic decision making. We implemented a crossover design in which all participants completed the decision tasks both with and without touch (in counterbalanced order), which allowed us to explore the effects both within and between subjects. Furthermore, we investigated betrayal aversion, altruism, and risk taking using three standard economic decision-making tasks: the Betrayal Aversion Elicitation Task (BAET), a dictator game, and the Balloon Analog Risk Task (BART).

## Materials and methods

### Participants

One hundred and twenty participants (43% female) were recruited from a subject pool at Linköping University, Sweden. Participants signed up using ORSEE (Greiner, 2015). Participants were Swedish-speaking students from a variety of disciplines. Ages ranged from 19 to 54 years (M = 24.8, SD = 6.0). A power calculation indicated that 101 participants were needed to detect a 0.25 effect size with 70% power within subjects. All participants gave written informed consent in accordance with the Declaration of Helsinki and were compensated with the amount earned on one randomly selected task. The procedures were approved by the regional ethics committee.

### Materials

#### Betrayal Aversion Elicitation Task (BAET)

**Figure 1:**
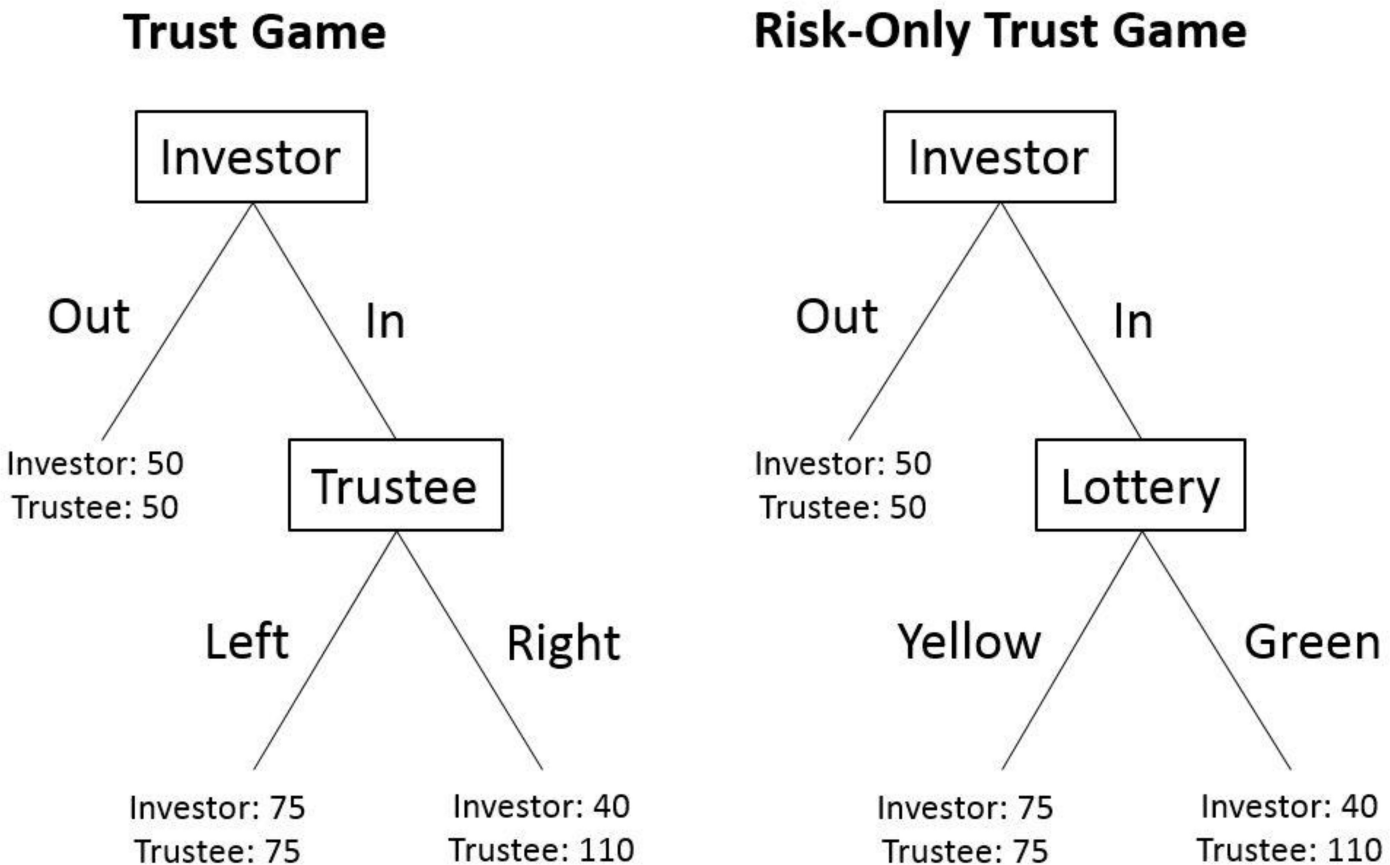
Structural overview of the Betrayal Aversion Elicitation Task, adapted from Aimone et al. (2015) and Quercia (2016).

Betrayal aversion was assessed using the Betrayal Aversion Elicitation Task (BAET; Aimone et al., 2015), which consists of two games: a trust game and a risk-only trust game (illustrated in Figure 1). In the trust game, the participant plays in the role of investor and is randomly paired with one other participant that plays in the role of trustee. The investor's task is to choose between *in* (trust) and *out* (don't trust). If they choose *out*, both the investor and the trustee receive 50 SEK (~6 USD). If they choose *in*, the amount they receive depends on the trustee's choice. The trustee chooses between *left* (reciprocate) and *right* (betray). If they choose *left*, both the trustee and the investor receive 75 SEK. If they choose *right*, the investor receives 40 SEK and the trustee receives 110 SEK.

All participants played in the role of investor. Prior to the study, a group of 20 participants completed the same trust game but in the role of trustee. That is, they indicated whether they would choose *left* or *right* if the investor chose *in*. The results from this part of the experiment determined investors' payoff. The investors' task was to indicate whether they chose *in* or *out*, for each possible value of the number of trustees that chose *left*. They made their decisions by filling out a choice list table consisting of 21 rows reporting all possible proportions of trustees choosing *left*, starting with “20 out of 20” in the first row and ending with “0 out of 20” in the last row. This elicitation method has been shown to increase participants' understanding of the task and to result in less noisy valuations, compared to an open-ended elicitation method (Quercia, 2016).

The risk-only trust game is identical to the trust game, except payoffs depend on a random lottery rather than on the trustee's decision. The lottery was described as an urn containing 20 colored balls that each can be either yellow or green. If a yellow ball is drawn, both the investor and the trustee receive 75 SEK. If a green ball is drawn, the investor receives 40 SEK and the trustee receives 110 SEK. The actual number of yellow and green balls was predetermined by the number of the 20 previous participants in the trust game who had chosen *left* and *right*, respectively. Thus, the probability of drawing a yellow ball in the risk-only trust game is the same as the probability of being paired with a trustee who chose *left* in the trust game.

The variable of interest in the BAET is the MAP of being paired with a trustee who chose *left* (in the trust game) or drawing a yellow ball (in the risk-only trust game) for which a participant is willing to choose *in*. We inferred each participant's MAP by calculating the mean between the last proportion for which they chose *in* and the first proportion for which they chose *out*, going from the top to the bottom of the choice list table^1^. Participants' betrayal aversion (BA) was then calculated as BA = MAP_TG_-MAP_ROTG_. If MAP_TG_ > MAP_ROTG_, participants are said to be betrayal averse. If MAP_TG_ < MAP_ROTG_, participants are said to be betrayal seeking. If MAP_TG_ = MAP_ROTG_, participants are said to be betrayal neutral.

#### Dictator game

Altruism was assessed using a dictator game in which participants distributed 100 SEK (~12 USD) between themselves and UNICEF. Participants indicated how much they wanted to keep for themselves and how much they wanted to give to UNICEF, using two sliding scales that ranged from 0 to 100 SEK, in 1 SEK increments. The sum of the scales had to equal 100 SEK.

#### Balloon Analog Risk Task (BART)

Risk taking was assessed using the Balloon Analog Risk Task (BART; Lejuez et al., 2002). On each of 30 trials, participants were presented with a picture of a balloon and were instructed that they could pump up the balloon to earn money. Each pump earned them 0.10 SEK. However, if they pumped up a balloon so much that it exploded, they earned 0 SEK on that trial. Risk taking is operationalized as the average number of pumps per trial, excluding trials on which the balloon exploded. We refer to this variable as the *adjusted average pumps*.

#### Self-report measures: touch pleasantness, game understanding, and attachment

Participants rated how pleasant and relaxing the touch was using two visual analog scales ranging from −10 (*very unpleasant/not relaxing at all*) to 10 (*very pleasant/very relaxing*). Game understanding was assessed following Quercia (2016; see Supplementary Materials)^2^. Attachment was assessed using the Revised Adult Attachment Scale (Collins, 1996), which consists of 18 items measuring how participants generally feel in important close relationships. The scale assesses both attachment avoidance and attachment anxiety. Participants indicated how characteristic each item was of them on a Likert-type scale from 1 (*not characteristic at all*) to 5 (*very characteristic*). Cronbach's alpha in our study was 0.84 for attachment avoidance and 0.86 for attachment anxiety. Finally, participants were asked to guess the purpose and hypotheses of the study and to report their suspicion of deception in the Betrayal Aversion Elicitation Task.

Complete instructions for all tasks are provided in the Supplementary Materials. All tasks except the BART were administered in Qualtrics. The BART was administered in Inquisit 5.

### Procedure

We implemented a crossover design in which participants performed the decision tasks twice: once in a touch condition and once in a no-touch control condition. The order of the tasks was the same for all participants—i.e., (1) Betrayal Aversion Elicitation Task, (2) dictator game, (3) Balloon Analog Risk Task—but the order of the touch and control conditions was counterbalanced between participants. Thus, participants served as their own controls.

Participants were seated at a desk equipped with a computer and were instructed to rest their left arm behind a curtain, palm facing down. The experimenter sat on the other side of the curtain. In the touch condition, the experimenter gently stroked the participant on the dorsal part of left forearm at a speed of 3 cm/s using a goat hair brush. This stroking procedure and velocity is optimal for activating CT fibers (Löken et al., 2009). The self-report measures confirmed that participants indeed perceived the touch as pleasant (M = 5.58, SD = 4.26) and relaxing (M = 3.92, SD = 5.11). The brushing began 60 s before the instructions for the first task were displayed and continued until completion of the last task. Thus, participants received touch both while reading the instructions for each task and while performing that task. In the control condition, participants received no touch, but the experimenter remained seated behind the curtain. Participants read the instructions for each task at their own pace immediately before completing that task. After completing all decision tasks twice (once in the touch condition and once in the control condition), participants filled out the self-report measures and were compensated for participating.

### Data analysis

We first investigated whether the proportion of participants who were classified as betrayal averse, betrayal neutral, and betrayal seeking differed between the touch and control condition, using a McNemar-Bowker test of symmetry. We then performed a paired samples *t*-test to investigate whether participants were on average less betrayal averse in the touch condition compared to the control condition. We also performed regression analyses in order to confirm the results from the *t*-test while controlling for factors such as age and gender. Our regression model was specified as follows:

$$\begin{array}{l} y_{ik}=\beta_{0}+\beta_{1}Touch+\beta_{2}Round+\beta_{4}X_{i}+\epsilon_{ik} \end{array}$$

where the dependent variable *y*_*ik*_ indicates the betrayal aversion (MAP_TG_-MAP_ROTG_) for participant *i* on round *k. Touch* is a dummy for the touch condition and *Round* is a dummy for the second round of the tasks, i.e., the second time participants performed the Betrayal Aversion Elicitation Task. **X**_*i*_ is the control variables age and gender. Alternative model (2) also included the interaction terms *Touch* × *Round*, which allows the effect of touch to differ across the two task rounds, and *Touch* × *Gender*, which allows the effect of touch to differ across genders. Alternative model (3) added the control variables attachment anxiety and attachment avoidance and alternative model (4) also included the interaction terms *Touch* × *Anxiety* and *Touch* × *Avoidance*, which allow the effect of touch to vary with attachment styles. The models were estimated using OLS and standard errors were corrected for clustering on the individual level.

Paired samples *t*-tests and regressions as specified above were also performed for altruism and risk taking, with mean amount donated to UNICEF and adjusted average pumps as dependent variables. We also investigated whether touch pleasantness correlated with betrayal aversion, altruism, and risk taking in the touch condition. Finally, we repeated all analyses using the corresponding between-subjects tests, to investigate the effect of touch in the first round of each task. The between-subjects analyses were performed because participants' responses are likely to be relatively consistent between the first and second round of the tasks and because the manipulation may be fairly obvious to participants, thus potentially influencing the results.

## Results

### The effect of touch on betrayal aversion

**Figure 2:**
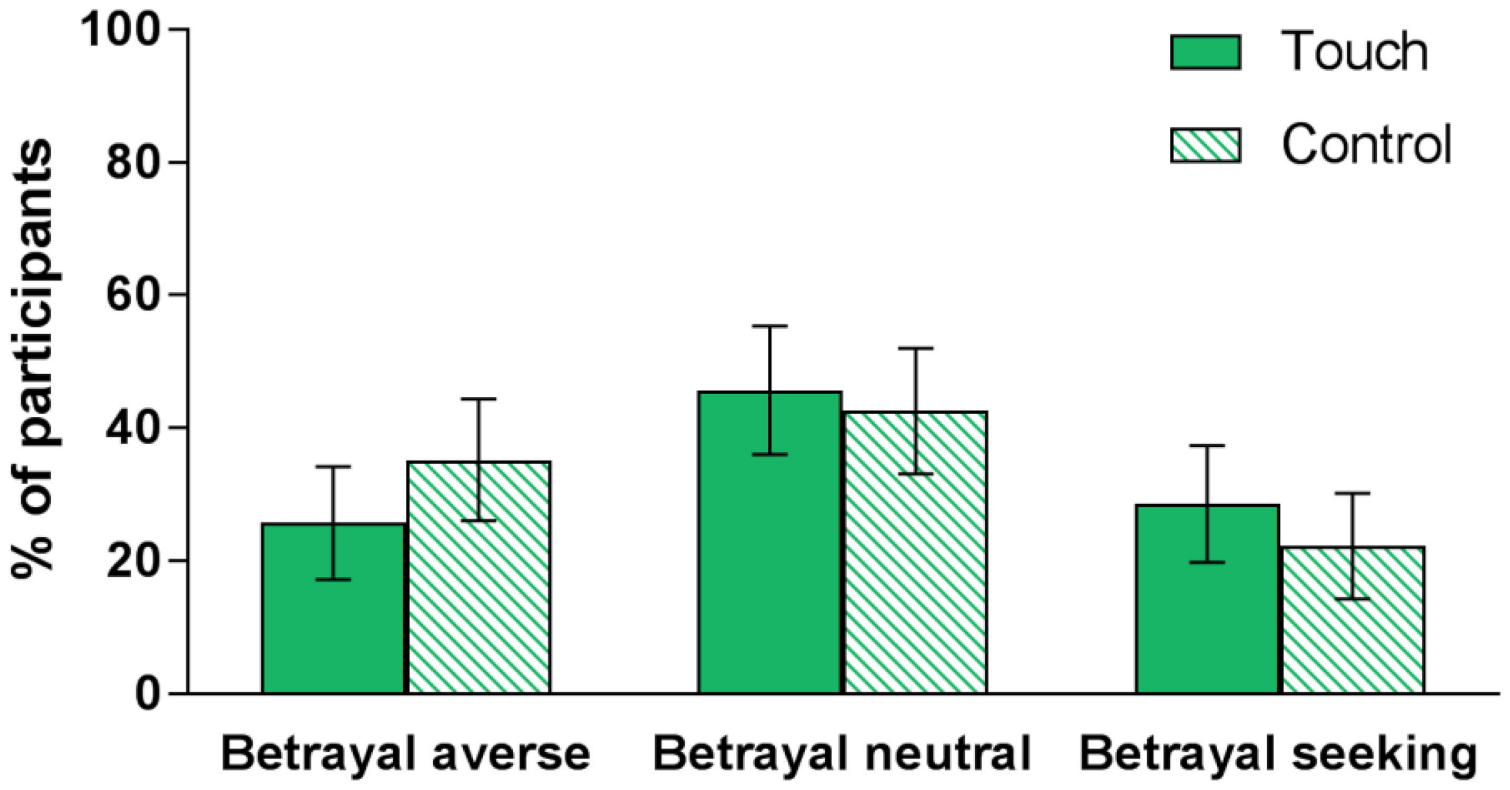
Proportion of participants in each condition (touch vs. control) who were classified as betrayal averse, betrayal neutral, and betrayal seeking. Error bars represent 95% confidence intervals.

Figure 2 displays the percentage of participants in each condition who were classified as betrayal averse (MAP_TG_ > MAP_ROTG_), betrayal neutral (MAP_TG_ = MAP_ROTG_), and betrayal seeking (MAP_TG_ < MAP_ROTG_). In the touch condition, 26% of participants were betrayal averse, 46% were betrayal neutral, and 29% were betrayal seeking. In the control condition, 35% of participants were betrayal averse, 43% were betrayal neutral, and 22% were betrayal seeking. Thus, participants were less betrayal averse in the touch condition. However, a McNemar-Bowker test of symmetry indicated that there was not a significant difference in the proportions of betrayal averse, betrayal neutral, and betrayal seeking participants between the touch and control conditions, *p* = 0.475^3^.

**Figure 3:**
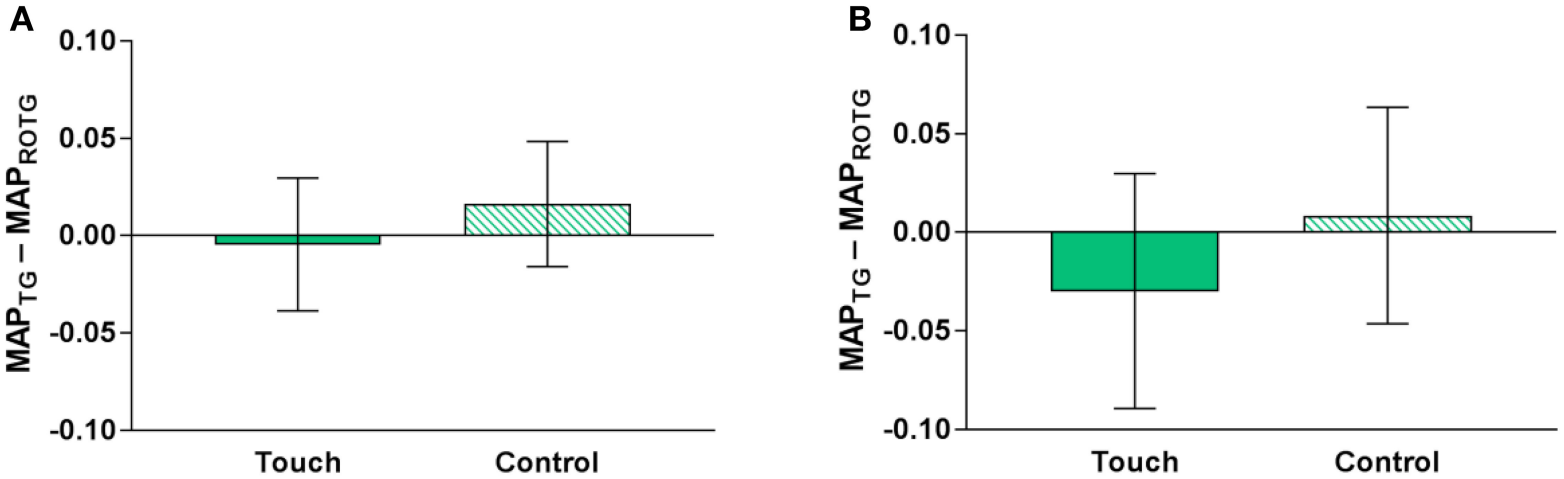
Betrayal aversion (MAP_TG_-MAP_ROTG_) in the touch and control conditions, **(A)** within subjects and **(B)** between subjects in the first round of the Betrayal Aversion Elicitation Task. Error bars represent 95% confidence intervals.

Figure 3A displays the average betrayal aversion (MAP_TG_-MAP_ROTG_) in the touch and control conditions (see also Supplementary Table 1). A paired samples *t*-test indicated that there was no significant difference in betrayal aversion between the two conditions, M_touch_ = −0.005 (95% CI [−0.039, 0.030]), M_control_ = 0.017 (95% CI [−0.016, 0.048]), *t*_(99)_ = −0.48, *p* = 0.633. The regression analyses found no significant effect either (see Table 1). That is, participants were not significantly less betrayal averse in the touch condition compared to the control condition, β = −0.021, *p* = 0.320. Touch pleasantness did not correlate with betrayal aversion in the touch condition, Spearman's rho = −0.09, *p* = 0.348.

**Table 1:** Regression analyses of betrayal aversion.

	**(1)**	**(2)**	**(3)**	**(4)**
Touch	−0.021	−0.017	−0.021	0.042
	(0.021)	(0.045)	(0.021)	(0.099)
Round	0.032	0.013	0.032	0.014
	(0.021)	(0.034)	(0.021)	(0.034)
Touch × Round		0.035		0.034
		(0.055)		(0.055)
Female	−0.012	0.013	−0.009	−0.009
	(0.026)	(0.036)	(0.027)	(0.027)
Touch × Female		−0.050		0.018
		(0.041)		(0.040)
Age	−0.001	−0.001	−0.001	−0.001
	(0.003)	(0.003)	(0.003)	(0.003)
Anxiety			−0.005	−0.004
			(0.013)	(0.014)
Touch × Anxiety				−0.002
				(0.017)
Avoidance			0.015	0.024
			(0.023)	(0.039)
Touch × Avoidance				−0.020
				(0.044)
Constant	0.022	0.023	−0.001	−0.028
	(0.071)	(0.075)	(0.099)	(0.134)

*This table reports OLS coefficient estimates (robust standard errors corrected for clustering on the individual level in parentheses). The dependent variable is participants' betrayal aversion (MAP_TG_-MAP_ROTG_). “Touch” is a dummy for the touch condition. “Round” is a dummy for the second round of the tasks, i.e., the second time the participants performed the tasks. “Touch × Round” is the interaction between the touch condition and the task round, allowing the effect of touch to differ across the two task rounds. “Female” is a gender dummy. “Touch × Female” is the interaction between the touch condition and gender, allowing the effect of touch to differ between men and women. “Age” is the participant's age in years. “Anxiety” is the participant's score on the attachment anxiety subscale. “Touch × Anxiety” is the interaction between the touch condition and attachment anxiety, allowing the effect of touch to vary with the level of attachment anxiety. “Avoidance” is the participant's score on the attachment avoidance subscale. “Touch × Avoidance” is the interaction between the touch condition and attachment avoidance, allowing the effect of touch to vary with the level of attachment avoidance. All ps > 0.10*.

Because participants' responses are likely to be relatively consistent between the first and second round of the task, we also performed a between-subjects analysis to investigate the effect of touch in the first round, i.e., the first time participants performed the task. Figure 3B displays the results from this analysis (see also Supplementary Table 2). An independent samples *t*-test indicated that there was no significant difference in betrayal aversion between the two conditions, M_touch_ = −0.030 (95% CI [−0.090, 0.030]), M_control_ = 0.008 (95% CI [−0.047, 0.064]), *t*_(102)_ = −0.95, *p* = 0.344. Regression analyses found no significant effects either (see Supplementary Table 2). There was a weak, negative correlation between betrayal aversion and touch pleasantness ratings, Spearman's rho = −0.28, *p* = 0.047. However, this correlation seemed to be driven by an outlier. When the outlier was excluded, the correlation was no longer significant, Spearman's rho = −0.24, *p* = 0.099.

### The effect of touch on altruism

**Figure 4:**
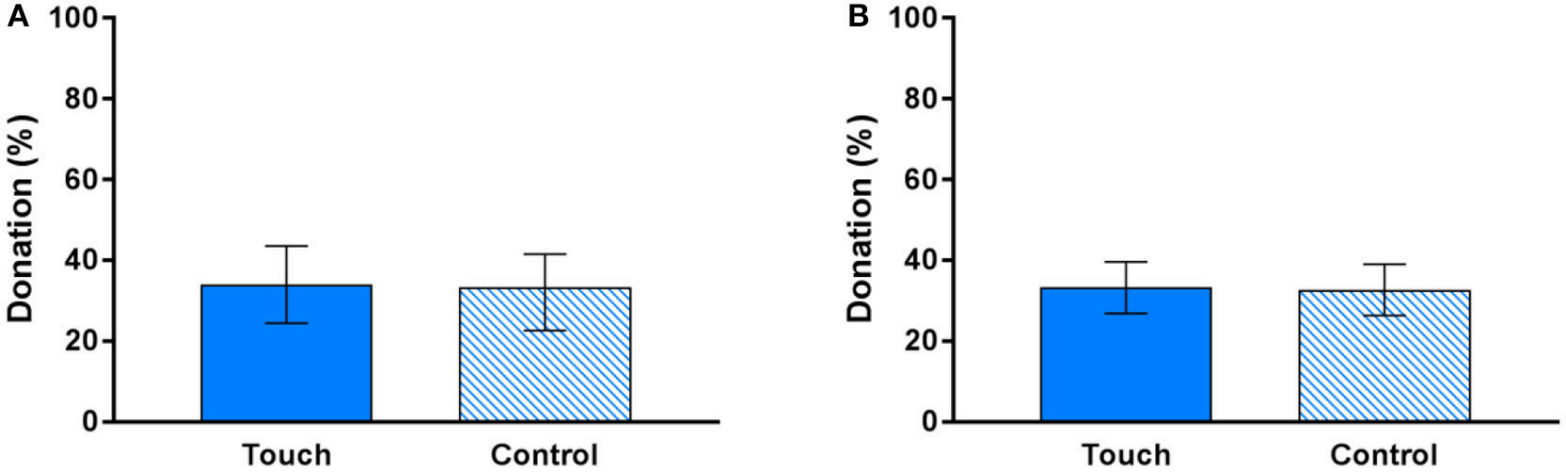
Donations to UNICEF in the touch and control conditions, **(A)** within subjects and **(B)** between subjects in the first round of the dictator game. Error bars represent 95% confidence intervals.

Figure 4A displays the mean amount donated to UNICEF in the dictator game, separated by condition (touch vs. control). There was no significant difference in donations between the two conditions, M_touch_ = 35.24% (95% CI [26.88, 39.62]), M_control_ = 32.70% (95% CI [26.34, 39.06]), paired samples *t*_(119)_ = 0.46, *p* = 0.649. The regression analyses found no significant effect either (see Table 2). That is, participants did not donate more money in the touch compared to the control condition, β = 0.550, *p* = 0.650. There was an interaction between touch and gender such that women donated more money to UNICEF in the touch than in the control condition; however, this interaction was only significant at the 10% level, β = 4.472, *p* = 0.072. Furthermore, there was a significant effect of attachment avoidance such that those high in attachment avoidance donated less money, β = −11.874, *p* = 0.014. However, this finding should be interpreted with caution since it is uncorrected for multiple hypothesis testing. Attachment anxiety had no significant effect and there were no interactions between touch and attachment anxiety or attachment avoidance. Touch pleasantness did not correlate with amount donated in the touch condition, Spearman's rho = 0.10, *p* = 0.256.

**Table 2:** Regression analyses of altruism.

	**(1)**	**(2)**	**(3)**	**(4)**
Touch	0.550	−0.501	0.550	−4.348
	(1.208)	(6.618)	(1.213)	(9.756)
Round	−1.383	0.422	−1.383	−0.794
	(1.208)	(6.517)	(1.213)	(6.386)
Touch × Round		−1.773		−1.086
		(12.822)		(12.571)
Female	2.103	−0.160	0.490	−1.795
	(6.347)	(6.469)	(6.637)	(6.772)
Touch × Female		4.472^*^		4.535^*^
		(2.462)		(2.596)
Age	0.780	0.787	0.877	0.881^*^
	(0.490)	(0.499)	(0.463)	(0.474)
Anxiety			−1.021	−1.219
			(3.587)	(3.642)
Touch × Anxiety				0.413
				(1.278)
Avoidance			−11.409	−11.874^**^
			(4.679)	(4.783)
Touch × Avoidance				0.936
				(1.627)
Constant	13.155	13.496	43.781^**^	46.086^**^
	(13.443)	(14.442)	(17.287)	(18.711)

*This table reports OLS coefficient estimates (robust standard errors corrected for clustering on the individual level in parentheses). The dependent variable is the amount donated to UNICEF. “Touch” is a dummy for the touch condition. “Round” is a dummy for the second round of the tasks, i.e., the second time the participants performed the tasks. “Touch × Round” is the interaction between the touch condition and the task round, allowing the effect of touch to differ across the two task rounds. “Female” is a gender dummy. “Touch × Female” is the interaction between the touch condition and gender, allowing the effect of touch to differ between men and women. “Age” is the participant's age in years. “Anxiety” is the participant's score on the attachment anxiety subscale. “Touch × Anxiety” is the interaction between the touch condition and attachment anxiety, allowing the effect of touch to vary with the level of attachment anxiety. “Avoidance” is the participant's score on the attachment avoidance subscale. “Touch × Avoidance” is the interaction between the touch condition and attachment avoidance, allowing the effect of touch to vary with the level of attachment avoidance*.

* *p < 0.10*,

** *p < 0.05*.

As with betrayal aversion, we also conducted between-subjects analyses to investigate the effect of touch in the first round. Figure 4B displays the mean amount donated to UNICEF in the first round of the dictator game, separated by condition. There was no significant difference between the two conditions, M_touch_ = 34.00% (95% CI [24.43, 43.57]), M_control_ = 33.33% (95% CI [24.55, 42.12]), independent samples *t*_(118)_ = 0.10, *p* = 0.918. Regression analyses found no significant effects either, apart from the effect of attachment avoidance mentioned above (see Supplementary Table 4). Touch pleasantness did not correlate with amount donated in the touch condition, Spearman's rho = −0.12, *p* = 0.343.

### The effect of touch on risk taking

**Figure 5:**
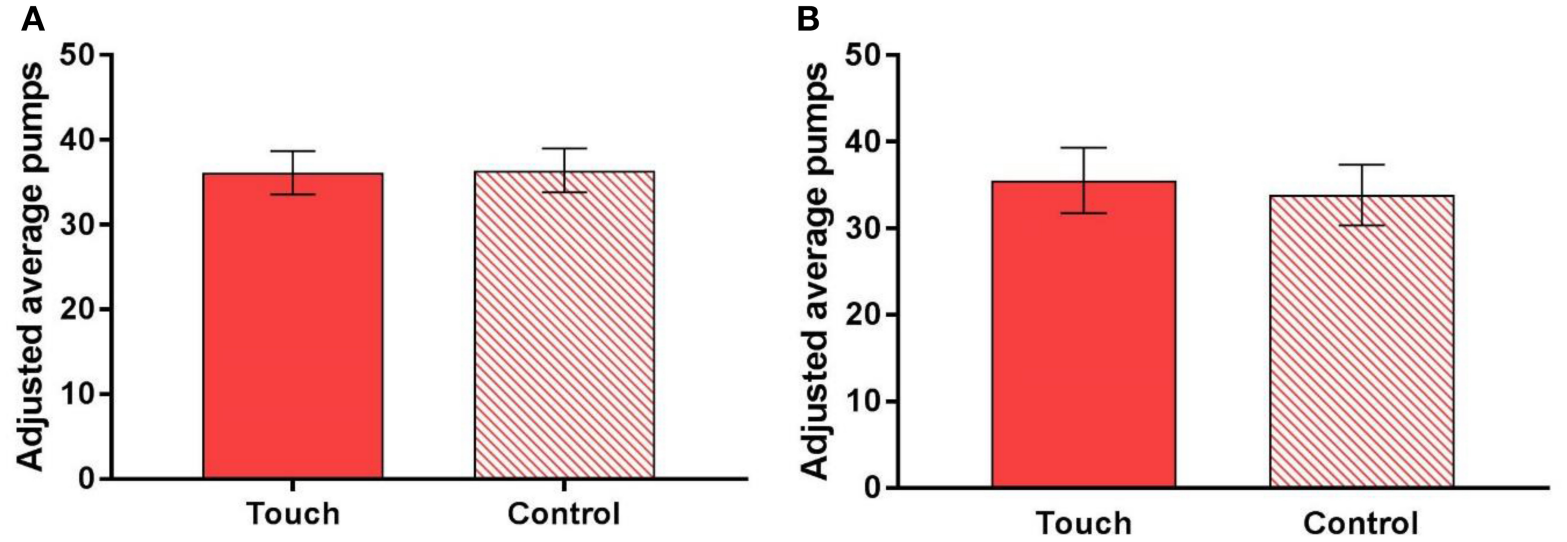
Average number of pumps per trial in the Balloon Analog Risk Task, excluding trials on which the balloon exploded and separated by condition (touch vs. control), **(A)** within subjects and **(B)** between subjects in the first round of the task. Error bars represent 95% confidence intervals.

Figure 5A displays the adjusted average number of pumps per trial in the BART, separated by condition (touch vs. control). There was no significant difference in the number of pumps between the two conditions, M_touch_ = 36.14 (95% CI [33.58, 38.69]), M_control_ = 36.40 (95% CI [33.83, 38.97]), paired samples *t*_(118)_ = −0.38, *p* = 0.708, thus indicating that affective touch does not influence risk taking^4^. The regression analyses found no significant effect either (see Table 3). That is, participants were not more risk taking in the touch condition compared to the control condition, β = −0.237, *p* = 0.714. However, there was a significant effect of *Round*, such that participants were more risk taking in the second compared to the first round of the tasks, β = 3.128, *p* < 0.0001. This is expected given that the number of pumps increases toward the end of the task (Lejuez et al., 2002). There was also a significant effect of gender, indicating that women were less risk taking than men, β = −7.487, *p* = 0.003. This is in line with previous findings from the BART (Lejuez et al., 2002) and from other measures of risk taking (Byrnes et al., 1999; Charness and Gneezy, 2012). Again, note that these *p*-values are uncorrected and should be interpreted with caution. Touch pleasantness did not correlate with risk taking in the touch condition, Spearman's rho = 0.08, *p* = 0.400.

**Table 3:** Regression analyses of risk taking.

	**(1)**	**(2)**	**(3)**	**(4)**
Touch	−0.237	1.814	−0.237	2.937
	(0.645)	(2.650)	(0.647)	(3.829)
Round	3.128^***^	5.179^**^	3.128^***^	5.268^**^
	(0.645)	(2.489)	(0.647)	(2.500)
Touch × Round		4.101		−4.289
		(4.848)		(4.860)
Female	−7.487^***^	−7.532^***^	−7.882^***^	−7.897^***^
	(2.421)	(2.519)	(2.473)	(2.539)
Touch × Female		−0.001		−0.102
		(1.297)		(1.328)
Age	−0.097	−0.082	−0.090	−0.074
	(0.172)	(0.167)	(0.175)	(0.171)
Anxiety			1.155	1.140
			(1.478)	(1.453)
Touch × Anxiety				0.143
				(0.815)
Avoidance			−1.171	−0.954
			(2.012)	(2.016)
Touch × Avoidance				−0.518
				(0.979)
Constant	40.488^***^	39.105^***^	40.622^***^	38.632^***^
	(4.542)	(4.588)	(6.864)	(6.918)

*This table reports OLS coefficient estimates (robust standard errors corrected for clustering on the individual level in parentheses). The dependent variable is adjusted average pumps, i.e., the average number of pumps per trial in the BART excluding trials on which the balloon exploded. “Touch” is a dummy for the touch condition. “Round” is a dummy for the second round of the tasks, i.e., the second time the participants performed the tasks. “Touch × Round” is the interaction between the touch condition and the task round, allowing the effect of touch to differ across the two task rounds. “Female” is a gender dummy. “Touch × Female” is the interaction between the touch condition and gender, allowing the effect of touch to differ between men and women. “Age” is the participant's age in years. “Anxiety” is the participant's score on the attachment anxiety subscale. “Touch × Anxiety” is the interaction between the touch condition and attachment anxiety, allowing the effect of touch to vary with the level of attachment anxiety. “Avoidance” is the participant's score on the attachment avoidance subscale. “Touch × Avoidance” is the interaction between the touch condition and attachment avoidance, allowing the effect of touch to vary with the level of attachment avoidance*.

** *p < 0.05*,

*** *p < 0.01*.

Figure 5B displays the adjusted average number of pumps per trial in the first round of the BART, separated by condition. There was no significant difference in the number of pumps between the two conditions, M_touch_ = 35.55 (95% CI [31.77, 39.33]), M_control_ = 33.85 (95% CI [30.34, 37.35]), paired samples *t*_(117)_ = 0.66, *p* = 0.510. Regression analyses found no significant effect either (see Supplementary Table 7). Touch pleasantness did not correlate with risk taking, Spearman's rho = 0.17, *p* = 0.203.

## Discussion

We investigated the effect of pleasant touch on betrayal aversion, altruism, and risk taking. Pleasant touch activates CT fibers in the skin, which are thought to mediate the oxytocin-enhancing effects of touch (Walker et al., 2017). Our results indicate no effect of touch on any of the outcome variables, neither within subjects nor between subjects. Furthermore, there were no significant interactions between touch and gender or attachment styles.

Given the lack of consistency in previous studies investigating the effect of oxytocin on trust (Nave et al., 2015), it is perhaps unsurprising that we find no effect of touch on betrayal aversion. Several issues have been pointed out in the oxytocin literature, including publication bias (Lane et al., 2016), low statistical power (Walum et al., 2016), lack of evidence that intranasal oxytocin reaches target brain areas (Leng and Ludwig, 2016), and unreliable measures of plasma oxytocin (McCullough et al., 2013; Christensen et al., 2014). This suggests that what we think we know about oxytocin in humans may not be true. Furthermore, as suggested by Bartz et al. (2011; see also Shamay-Tsoory and Abu-Akel, 2016), the effect of oxytocin on trust and prosocial behavior—if there is one—is likely constrained by both individual and contextual factors. For example, previous studies have suggested that oxytocin reduces investments in a trust game following betrayal in women but not in men (Yao et al., 2014) and that oxytocin increases trust and reduces betrayal aversion in individuals that are high, compared to low, in attachment avoidance (De Dreu, 2012). However, in our study, we found no significant interactions between touch and gender or attachment. Regarding contextual factors, previous studies have shown that oxytocin increases trust when trustees are described as trustworthy but not when they are described as untrustworthy (Mikolajczak et al., 2010) and that oxytocin increases trust and altruism toward the in-group but results in defensive behaviors toward the outgroup (De Dreu et al., 2010). Oxytocin also increased cooperation (which requires some degree of trust) in a coordination game when there had been prior contact between participants but reduced cooperation when there had been no prior contact (Declerck et al., 2010). This finding is particularly noteworthy because in the study by Kosfeld et al. (2005), which provided the initial evidence for a causal link between oxytocin and trust, participants introduced themselves to each other before they played the trust game. In contrast, participants in our study played with anonymous counterparts. Therefore, given that oxytocin may enhance the salience of social cues (Shamay-Tsoory and Abu-Akel, 2016), the absence of an effect of touch on betrayal aversion in our study could, at least in part, be due to the lack of social information.

An alternative explanation for our null effect of touch on betrayal aversion is that the size of betrayal aversion was small to begin with, indicating that participants made little difference between the trust game and the risk-only trust game. Early studies reported betrayal aversion sizes ranging from 0.08 to 0.22 (Bohnet et al., 2008). Studies using the Betrayal Aversion Elicitation Task, which assesses betrayal aversion within subjects, have reported betrayal aversion sizes of 0.04 (Aimone et al., 2015) and 0.07 (Quercia, 2016). Betrayal aversion in our study was −0.005 (indicating slightly betrayal seeking or betrayal neutral) in the touch condition and 0.017 (slightly betrayal averse) in the control condition. Furthermore, the proportion of participants that could be categorized as betrayal averse was lower than the proportion of participants that could be categorized as betrayal neutral, which contradicts previous findings that people generally are betrayal averse (Bohnet and Zeckhauser, 2004; Bohnet et al., 2008; Aimone et al., 2015). One possible explanation for these discrepancies is that participants in previous studies (e.g., Bohnet and Zeckhauser, 2004; Bohnet et al., 2008; Aimone et al., 2015; Quercia, 2016) were tested in groups, meaning that any betrayal occurred there and then as a result of the decision of another participant that was present in the same room. In contrast, participants in our study were tested individually and played with anonymous counterparts who had already made their decisions prior to the study. Therefore, the potential betrayal may have felt less personal, which, in turn, may have reduced the negative affective experience associated with the possibility of being betrayed (Lauharatanahirun et al., 2012; Aimone et al., 2014).

The reason we found no effect of touch on altruism could, again, be that there is no direct, causal effect and/or that it depends on individual and contextual factors. Some researchers have found that oxytocin increases donations to charitable organizations (Barraza et al., 2011; Marsh et al., 2015) and that it increases monetary contributions both to the in-group and to the outgroup in a social dilemma (Israel et al., 2012). The prediction that follows from this line of research is that touch increases altruism, which is not what we found in the present study. Instead, our findings are in line with studies showing no effect of oxytocin on altruism (Zak et al., 2007; Hu et al., 2016). Nevertheless, other researchers have found that the effect depends on the closeness of the relationship to the target (Pornpattananangkul et al., 2017) and whether the target belongs to the in-group or the outgroup (De Dreu et al., 2010). We did not take such contextual factors into account. We did find a trend such that touch increased altruism in women more than in men, but this interaction was significant only at the 10% significance level and should be interpreted with caution.

The lack of an effect of touch on risk taking makes sense given that previous studies have found no effect of oxytocin on non-social risk taking (e.g., Kosfeld et al., 2005; Patel et al., 2015). However, it is at odds with studies showing that brief physical contact increases risk taking (Levav and Argo, 2010). A limitation of our study is that we did not measure actual hormone levels, so we cannot rule out the possibility that our lack of effects is due to a failure to increase oxytocin. An alternative possibility is that touch increases positive affect, which, in turn, reduces betrayal aversion and increases altruism and risk taking. Indeed, CT-optimal touch is perceived as pleasant and rewarding (Perini et al., 2015) and positive affect has been suggested as one of the mechanisms underlying the Midas effect (Schirmer et al., 2016). However, studies finding an effect of touch on altruism and risk taking have investigated touch in the form of brief physical contact, such as a tap on the shoulder (Kleinke, 1977; Crusco and Wetzel, 1984; Guéguen and Fischer-lokou, 2003; Levav and Argo, 2010). Here, we investigated the effect of continuous, gentle stroking that lasted throughout the decision phase. This distinction is important for several reasons. First, it is possible that any effect in our study was reduced because the manipulation was obvious to participants. Second, it is possible that participants attributed any affective changes to the touch and that the influence on behavior diminished as a result. Third, incidental affect from the touch may not have been strong enough to override integral affect from the BART, which is rich in affective cues (for a discussion of the integration of incidental and integral affect in decision making, see Västfjäll et al., 2016). Moreover, in studies reporting an effect of brief touch on prosocial behavior, the prosocial behavior was directed toward the toucher, such as the waitress receiving a tip (Crusco and Wetzel, 1984). In contrast, participants in our study donated money to a charitable organization. It is possible that increases in positive affect are attributed to the person delivering the touch, and that touch therefore promotes prosocial behavior only toward the toucher.

In conclusion, we found no effect of touch on betrayal aversion, altruism, or risk taking. These results add to a growing body of research suggesting that oxytocin has no direct, causal effect on trust and prosocial behaviors. Nonetheless, we remain optimistic that touch plays a vital role for social and psychological well-being. It is possible that its effects on economic decision making and behavior are dependent on the social context in a way that may be difficult to study in a laboratory setting. Future research should continue to investigate the circumstances under which affective touch—and its hormonal correlates—influences social behaviors and economic decision making.

## Ethics statement

This study was carried out in accordance with the recommendations of human research guidelines, Regional Ethics Board for East Gothland with written informed consent from all subjects.

## Author contributions

LK, IM, DV, and GT developed the study concept and design. LK collected the data and LK and DA analyzed the results. All authors contributed to the interpretation of the data. LK wrote the first draft of the paper and DA, IM, DV, and GT provided revisions. All authors approved the final version.

### Conflict of interest statement

The authors declare that the research was conducted in the absence of any commercial or financial relationships that could be construed as a potential conflict of interest.

## Footnotes

^1^We did not force participants to switch between *in* and *out* only once in the choice list table. As a result, 6–9% of participants in each task had multiple switching points in their responses. In two of these cases, the switching points occurred in the middle of the table, allowing us to infer the participant's MAP by taking the average between the first and the last switching point. The rest of the participants with multiple switching points were excluded from the analysis. Participants were also excluded if they selected *out* in the first row and switched to *in* at some point in the table. If a participant chose *in* for all rows of the table, their MAP was set to 0; if they chose *out* for all rows, it was set to 1.

^2^Contrary to Quercia (2016) and Aimone et al. (2015), we did not assess game understanding immediately following the instructions and before participants filled in the choice list table, because we wanted to avoid priming analytical, “system 2” thinking. Leaving the comprehension questions to the end of the experiment also ensured that the two rounds of the task were identical to the greatest extent possible. We assume that participants understood the task if we were able to infer a MAP from their responses. Results from the comprehension questions are reported in the Supplementary Materials and are similar to those reported in Quercia (2016).

^3^The difference in proportions of betrayal averse, betrayal neutral, and betrayal seeking participants in the first round of the Betrayal Aversion Elicitation Task was also non-significant, Chi-Square test, *p* = 0.727 (see Supplementary Figure 1).

^4^One participant's data was lost due to technical issues. Separating the task into the first, middle, and last 10 trials yielded no significant results (see Supplementary Table 5, 6).
