# Supplementary material for: The (Null) Effect of Affective Touch on Betrayal Aversion, Altruism, and Risk Taking
Source: Front Behav Neurosci. 2017 Dec 19;11:251. doi: 10.3389/fnbeh.2017.00251 (PMC5742217; doi:10.3389/fnbeh.2017.00251)
Supplement: Supplementary file 1 [file DataSheet1.docx]

***Supplementary Material***

**The (Null) Effect of Affective Touch on Betrayal Aversion, Altruism, and Risk Taking**

**Lina Koppel, David Andersson, India Morrison, Daniel Västfjäll, Gustav Tinghög**

*Correspondence: Gustav Tinghög, [gustav.tinghog@liu.se](mailto:gustav.tinghog@liu.se)

**
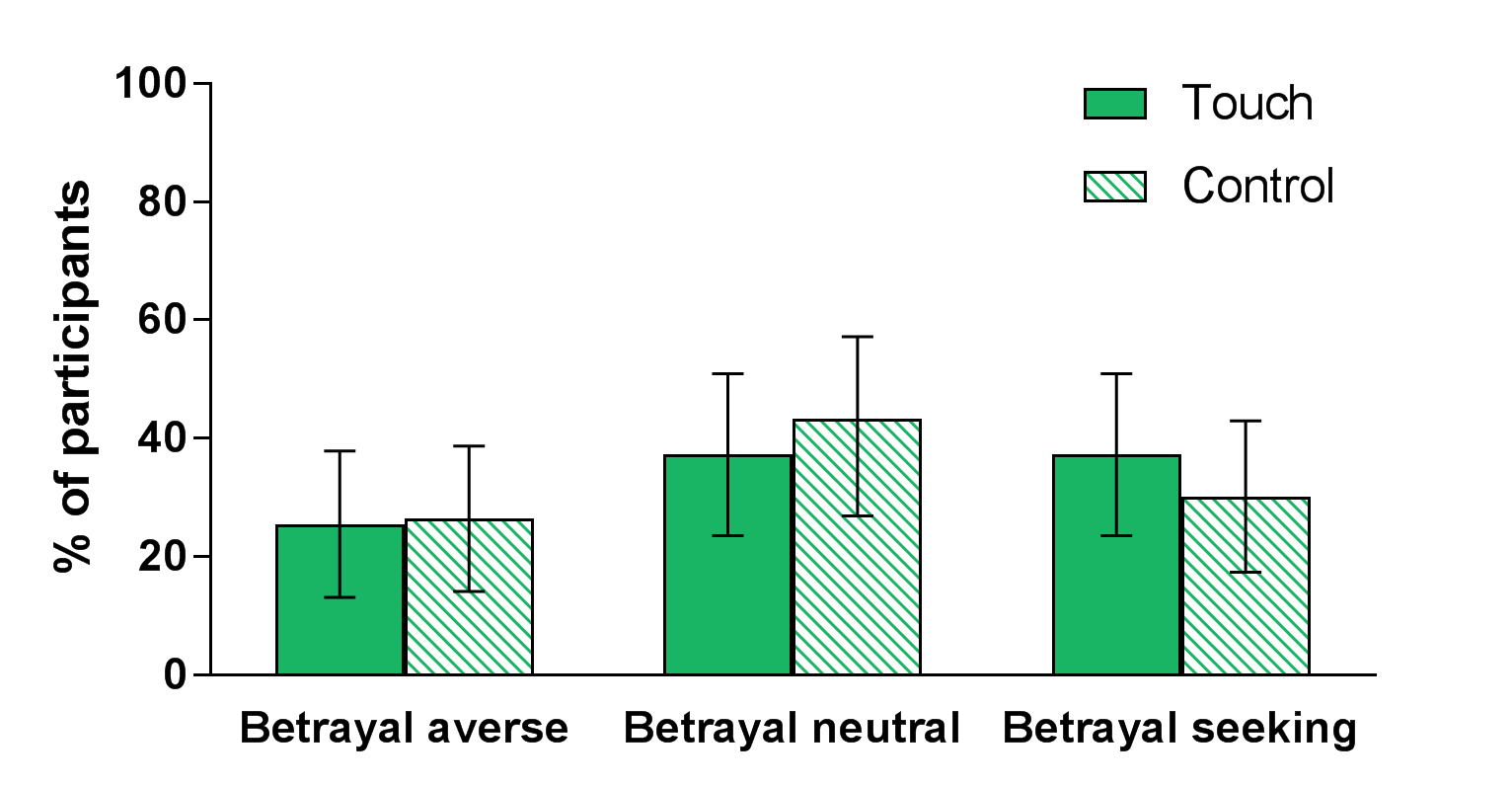
**

**Supplementary Figure 1.** Proportion of participants in each condition (touch vs. control) who were classified as betrayal averse, betrayal neutral, and betrayal seeking in the first round of the Betrayal Aversion Elicitation Task. Error bars represent 95% confidence intervals.

**Supplementary Table 1.** Betrayal aversion, altruism, and risk taking in each condition, before and after data exclusions (mean [95% CI])

|  | Condition | |  | Paired samples t-test | |
| --- | --- | --- | --- | --- | --- |
|  | Touch | Control |  | *df* | *p* |
| **Exclude: none** | | | | | |
| MAP_TG_ | 0.43 [0.39, 0.48] | 0.44 [0.40, 0.49] |  | 103 | .347 |
| MAP_ROTG_ | 0.40 [0.35, 0.44] | 0.40 [0.35, 0.44] |  | 108 | .859 |
| MAP_TG_ – MAP_ROTG_ | –0.00 [0.04, 0.03] | 0.02 [–0.02, 0.05] |  | 99 | .633 |
| Donation (%) | 35.24 [26.88, 39.62] | 32.70 [26.34, 39.06] |  | 119 | .649 |
| BART_adjusted_ | 36.14 [33.58, 38.69] | 36.40 [33.83, 38.97] |  | 118 | .708 |
| **Exclude: touch pleasantness ≤ 0** | | | | | |
| MAP_TG_ | 0.43 [0.38, 0.48] | 0.45 [0.40, 0.50] |  | 83 | .193 |
| MAP_ROTG_ | 0.41 [0.36, 0.46] | 0.40 [0.35, 0.45] |  | 88 | .421 |
| MAP_TG_ – MAP_ROTG_ | –0.02 [–0.06, 0.02] | 0.02 [–0.01, 0.06] |  | 80 | .143 |
| Donation (%) | 32.24 [25.55, 38.94] | 32.90 [26.05, 39.74] |  | 97 | .504 |
| BART_adjusted_ | 36.57 [33.79, 39.36] | 36.96 [34.10, 39.82] |  | 97 | .629 |
| **Exclude: MAP = 0 or MAP = 1** | | | | | |
| MAP_TG_ | 0.46 [0.42, 0.50] | 0.46 [0.42, 0.50] |  | 90 | .908 |
| MAP_ROTG_ | 0.44 [0.40, 0.49] | 0.44 [0.40, 0.48] |  | 95 | .872 |
| MAP_TG_ – MAP_ROTG_ | –0.00 [–0.03, 0.03] | 0.00 [–0.02, 0.02] |  | 87 | .812 |
| **Exclude: touch pleasantness ≤ 0, MAP = 0, or MAP = 1** | | | | | |
| MAP_TG_ | 0.46 [0.42, 0.51] | 0.47 [0.42, 0.52] |  | 73 | .840 |
| MAP_ROTG_ | 0.45 [0.41, 0.50] | 0.45 [0.40, 0.49] |  | 78 | .690 |
| MAP_TG_ – MAP_ROTG_ | –0.01 [–0.04, 0.02] | –0.00 [–0.03, 0.03] |  | 71 | .504 |

**Supplementary Table 2.** Betrayal aversion, altruism, and risk taking in each condition in the first round of each task, before and after data exclusions (mean [95% CI])

|  | Condition | |  | Independent  samples t-test | | |
| --- | --- | --- | --- | --- | --- | --- |
|  | Touch | Control |  | *df* | *p* |  |
| **Exclude: none** | | | | | | |
| MAP_TG_ | 0.43 [0.36, 0.50] | 0.45 [0.38, 0.51] |  | 106 | .797 |  |
| MAP_ROTG_ | 0.44 [0.37, 0.50] | 0.43 [0.37, 0.50] |  | 111 | .914 |  |
| MAP_TG_ – MAP_ROTG_ | –0.03 [–0.09,0.03] | 0.01 [–0.05, 0.06] |  | 102 | .344 |  |
| Donation (%) | 34.00 [24.43, 43.57] | 33.33 [24.55, 42.12] |  | 118 | .918 |  |
| BART_adjusted_ | 35.55 [31.77, 39.33] | 33.85 [30.34, 37.35] |  | 117 | .510 |  |
| **Exclude: touch pleasantness ≤ 0** | | | | | | |
| MAP_TG_ | 0.44 [0.37, 0.51] | 0.44 [0.37, 0.52] |  | 86 | .908 |  |
| MAP_ROTG_ | 0.45 [0.38, 0.52] | 0.42 [0.35, 0.49] |  | 89 | .552 |  |
| MAP_TG_ – MAP_ROTG_ | –0.04 [–0.11, 0.03] | 0.02 [–0.05, 0.09] |  | 82 | .199 |  |
| Donation (%) | 33.92 [23.66, 44.18] | 32.13 [22.96, 41.30] |  | 96 | .795 |  |
| BART_adjusted_ | 35.85 [32.04, 39.66] | 34.18 [29.94, 38.42] |  | 96 | .555 |  |
| **Exclude: MAP = 0 or MAP = 1** | | | | | | |
| MAP_TG_ | 0.46 [0.39, 0.52] | 0.46 [0.40, 0.52] |  | 92 | .975 |  |
| MAP_ROTG_ | 0.46 [0.41, 0.52] | 0.46 [0.40, 0.52] |  | 97 | .946 |  |
| MAP_TG_ – MAP_ROTG_ | –0.02 [–0.07, 0.03] | –0.01 [–0.04, 0.01] |  | 89 | .917 |  |
| **Exclude: touch pleasantness ≤ 0, MAP = 0, or MAP = 1** | | | | | | |
| MAP_TG_ | 0.46 [0.39, 0.52] | 0.46 [0.39, 0.54] |  | 75 | .887 |  |
| MAP_ROTG_ | 0.47 [0.41, 0.54] | 0.47 [0.40, 0.54] |  | 78 | .977 |  |
| MAP_TG_ – MAP_ROTG_ | –0.03 [–0.08, 0.03] | –0.02 [–0.05, 0.01] |  | 72 | .897 |  |

**Supplementary Table 3.** Regression analyses of betrayal aversion in the first round of the Betrayal Aversion Elicitation Task

|  | (1) | (2) | (3) | (4) |
| --- | --- | --- | --- | --- |
| Touch | –0.039  (0.042) | –0.007  (0.053) | –0.039  (0.042) | 0.143  (0.169) |
| Female | 0.006  (0.043) | 0.045  (0.058) | 0.008  (0.048) | 0.039  (0.070) |
| Touch × Female |  | –0.079  (0.072) |  | –0.075  (0.080) |
| Age | –0.001  (0.005) | –0.002  (0.005) | –0.001  (0.005) | –0.002  (0.005) |
| Anxiety |  |  | –0.001  (0.017) | 0.022  (0.023) |
| Touch × Anxiety |  |  |  | –0.052  (0.036) |
| Avoidance |  |  | 0.011  (0.041) | 0.017  (0.070) |
| Touch × Avoidance |  |  |  | –0.007  (0.075) |
| Constant | 0.026  (0.128) | 0.030  (0.128) | 0.002  (0.187) | –0.067  (0.229) |

*Notes.* This table reports OLS coefficient estimates (robust standard errors corrected for clustering on the individual level in parentheses). The dependent variable is participants’ betrayal aversion (MAP_TG_ – MAP_ROTG_) in the first round of the Betrayal Aversion Elicitation Task. “Touch” is a dummy for the touch condition. “Female” is a gender dummy. “Touch × Female” is the interaction between the touch condition and gender, allowing the effect of touch to differ between men and women. “Age” is the participant’s age in years. “Anxiety” is the participant’s score on the attachment anxiety subscale. “Touch × Anxiety” is the interaction between the touch condition and attachment anxiety, allowing the effect of touch to vary with the level of attachment anxiety. “Avoidance” is the participant’s score on the attachment avoidance subscale. “Touch × Avoidance” is the interaction between the touch condition and attachment avoidance, allowing the effect of touch to vary with the level of attachment avoidance. All *p*s > .10.

**Supplementary Table 4.** Regression analyses of altruism in the first round of the dictator game

|  | (1) | (2) | (3) | (4) |
| --- | --- | --- | --- | --- |
| Touch | 1.381  (6.555) | 2.674  (8.802) | 0.962  (6.386) | 3.098  (29.012) |
| Female | 2.462  (6.529) | 3.986  (8.670) | 0.828  (6.789) | 2.638  (9.646) |
| Touch × Female |  | –3.049  (12.928) |  | –4.179  (14.072) |
| Age | 0.746  (0.503) | 0.720  (0.499) | 0.851  (0.473) | 0.839  (0.498) |
| Anxiety |  |  | –1.774  (3.624) | 0.180  (4.924) |
| Touch × Anxiety |  |  |  | –4.454  (7.424) |
| Avoidance |  |  | –12.640***  (4.761) | –14.613**  (6.555) |
| Touch × Avoidance |  |  |  | 4.141  (9.791) |
| Constant | 13.427  (14.594) | 13.446  (14.655) | 49.148**  (18.970) | 48.942**  (22.334) |

*Notes.* This table reports OLS coefficient estimates (robust standard errors corrected for clustering on the individual level in parentheses). The dependent variable is the amount donated to UNICEF in the first round of the dictator game. “Touch” is a dummy for the touch condition. “Female” is a gender dummy. “Touch × Female” is the interaction between the touch condition and gender, allowing the effect of touch to differ between men and women. “Age” is the participant’s age in years. “Anxiety” is the participant’s score on the attachment anxiety subscale. “Touch × Anxiety” is the interaction between the touch condition and attachment anxiety, allowing the effect of touch to vary with the level of attachment anxiety. “Avoidance” is the participant’s score on the attachment avoidance subscale. “Touch × Avoidance” is the interaction between the touch condition and attachment avoidance, allowing the effect of touch to vary with the level of attachment avoidance.

* *p* < .10, ** *p* < .05, *** *p* < .01

**Supplementary Table 5.** Adjusted average number of pumps per trial in the Balloon Analog Risk Task, separated by the first, middle, and last 10 trials

|  | Condition | |  | Paired samples t-test |
| --- | --- | --- | --- | --- |
|  | Touch | Control |  | *p* |
| First 10 | 34.10 [31.36, 36.85] | 34.50 [31.53, 37.46] |  | .728 |
| Middle 10 | 36.33 [33.50, 39.15] | 37.85 [34.96, 40.74] |  | .126 |
| Last 10 | 37.72 [35.16, 40.28] | 37.20 [34.62, 39.79] |  | .525 |

**Supplementary Table 6.** Adjusted average number of pumps per trial in the first round of the Balloon Analog Risk Task, separated by the first, middle, and last 10 trials

|  | Condition | |  | Paired samples t-test |
| --- | --- | --- | --- | --- |
|  | Touch | Control |  | *p* |
| First 10 | 32.40 [28.20, 36.60] | 29.83 [26.01, 33.65] |  | .368 |
| Middle 10 | 36.72 [32.28, 41.15] | 35.44 [31.61, 39.27] |  | .663 |
| Last 10 | 37.10 [33.41, 40.79] | 36.59 [32.75, 40.44] |  | .849 |

**Supplementary Table 7.** Regression analyses of risk taking in the first round of the Balloon Analog Risk Task

|  | (1) | (2) | (3) | (4) |
| --- | --- | --- | --- | --- |
| Touch | 1.688  (2.529) | 3.898  (3.490) | 1.761  (2.546) | 11.740  (11.774) |
| Female | –6.994***  (2.503) | –4.396  (3.579) | –7.355***  (2.583) | –5.102  (3.696) |
| Touch × Female |  | –5.166  (5.219) |  | –4.980  (5.612) |
| Age | –0.185  (0.173) | –0.229  (0.177) | –0.176  (0.175) | –0.235  (0.194) |
| Anxiety |  |  | 0.890  (1.483) | 1.551  (1.929) |
| Touch × Anxiety |  |  |  | –1.632  (2.974) |
| Avoidance |  |  | –1.317  (2.083) | –0.493  (2.895) |
| Touch × Avoidance |  |  |  | –1.457  (4.371) |
| Constant | 41.500***  (4.734) | 41.503***  (4.709) | 45.568***  (6.878) | 39.286***  (8.304) |

*Notes.* This table reports OLS coefficient estimates (robust standard errors corrected for clustering on the individual level in parentheses). The dependent variable is adjusted average pumps in the first round of the BART, i.e., the average number of pumps per trial excluding trials on which the balloon exploded. “Touch” is a dummy for the touch condition. “Female” is a gender dummy. “Touch × Female” is the interaction between the touch condition and gender, allowing the effect of touch to differ between men and women. “Age” is the participant’s age in years. “Anxiety” is the participant’s score on the attachment anxiety subscale. “Touch × Anxiety” is the interaction between the touch condition and attachment anxiety, allowing the effect of touch to vary with the level of attachment anxiety. “Avoidance” is the participant’s score on the attachment avoidance subscale. “Touch × Avoidance” is the interaction between the touch condition and attachment avoidance, allowing the effect of touch to vary with the level of attachment avoidance.

* *p* < .10, ** *p* < .05, *** *p* < .01

**Supplementary Table 8.** Answers to comprehension questions

|  | Rating |  | Wrong answers |
| --- | --- | --- | --- |
|  | *M* (*SD*) |  | % |
| **Question 1:** Difficulty of instructions | 3.78 (2.18) |  |  |
| **Question 2** |  |  |  |
| a) Would payoffs depend on counterpart’s decision? |  |  | 7.5% |
| b) Your payoff |  |  | 5.8% |
| c) Counterpart’s payoff |  |  | 4.2% |
| **Question 3** |  |  |  |
| a) Would payoffs depend on counterpart’s decision? |  |  | 25.0% |
| b) Your payoff |  |  | 39.2% |
| c) Counterpart’s payoff |  |  | 42.5% |

**Instructions for the Betrayal Aversion Elicitation Task**

**INSTRUCTIONS TASK 1**

**Basic structure of the game**

In this game, you will be randomly paired with one other participant, your counterpart.

You choose between two alternatives, **In** and **Out**. If you choose **Out**, you and your counterpart get 50 SEK each. If you choose **In**, the amount you get depends on your counterpart’s decision.

Your counterpart chooses between **Left** and **Right**. If he/she chooses **Left**, you get 75 SEK each. If he/she chooses **Right**, you get 40 SEK and your counterpart gets 110 SEK.


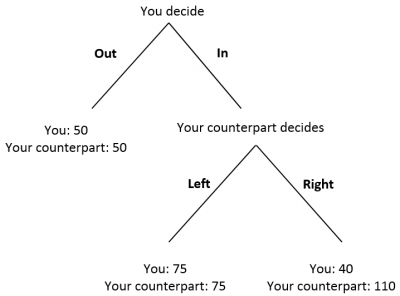


**Your task**

Before you arrived to the experiment, we let 20 participants play the game described above. They all played in the role of your counterpart. That is, they answered the question: "Do you choose **Left** or **Right** if the person you are paired with chooses **In**?"

It is now your turn to play. Your task is to consider not only the possible action of your counterpart but also the possible actions of all 20 participants. In other words, your task is to decide between **In** and **Out** *for all possible values of the number of participants who chose****Left***. That is: Do you choose **In** or **Out** if 20 of 20 participants chose **Left**? Do you choose **In** or **Out** if 19 of 20 participants chose **Left**? Do you choose **In** or **Out** if 18 of 20 participants chose **Left**? And so on.

You will make your decisions by filling in a table similar to the one below:


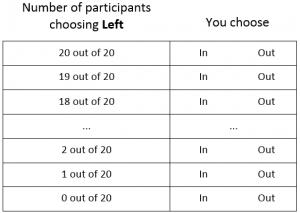


Select either **In** or **Out** for each row of the table.

**How payment is calculated**

At the end of the experiment, we will count how many of the 20 previous participants chose **Left**. This will indicate which row of the table we will look at to determine your (and your counterpart's) payment.

**EXAMPLE 1:** Suppose that 19 out of 20 participants chose **Left**. We will then select row 2 of the table you completed. Suppose further that you chose **In** in that row. Then, **In** would be the decision of yours that is relevant to your earnings. At that point, there would be two possible cases: either your counterpart is one of the 19 participants who chose **Left** or he/she is the one who chose **Right**. In the former case, you and your counterpart get 75 SEK each. In the latter case, you get 40 SEK and your counterpart gets 110 SEK.

**EXAMPLE 2:** Suppose that 1 out of 20 participants chose **Left** and that you chose **Out** in the corresponding row. Then, **Out** will be the decision of yours that is relevant to your earnings. In this case, you and your counterpart get 50 SEK each, regardless of whether your counterpart chose **Left** or **Right**.

You will be paid in cash right after you finish the experiment. Your counterpart will be contacted via e-mail and will be asked to collect his/her payment. We will not reveal your identity to your counterpart or your counterpart's identity to you.

Remember: all the decisions you take are potentially important because you don’t yet know the actual number of participants who chose **Left**.

Click on the arrow to begin.

[Page break]


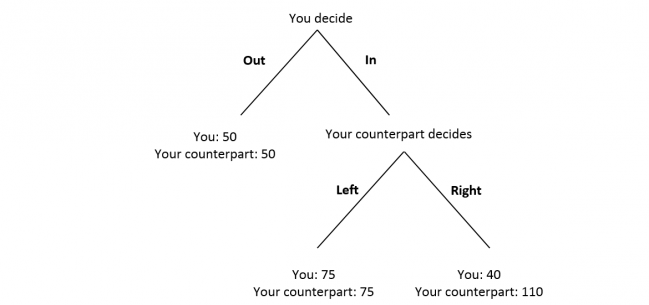


Number of participants choosing **Left**: You choose:

|  | In | Out |
| --- | --- | --- |
| 20 out of 20 |  |  |
| 19 out of 20 |  |  |
| 18 out of 20 |  |  |
| 17 out of 20 |  |  |
| 16 out of 20 |  |  |
| 15 out of 20 |  |  |
| 14 out of 20 |  |  |
| 13 out of 20 |  |  |
| 12 out of 20 |  |  |
| 11 out of 20 |  |  |
| 10 out of 20 |  |  |
| 9 out of 20 |  |  |
| 8 out of 20 |  |  |
| 7 out of 20 |  |  |
| 6 out of 20 |  |  |
| 5 out of 20 |  |  |
| 4 out of 20 |  |  |
| 3 out of 20 |  |  |
| 2 out of 20 |  |  |
| 1 out of 20 |  |  |
| 0 out of 20 |  |  |

[Page break]

**INSTRUCTIONS TASK 2**

**Basic structure of the game**

In this game, you will be randomly paired with one other participant, your counterpart.

You choose between two alternatives, **In** and **Out**. If you choose **Out**, you and your counterpart get 50 SEK each. If you choose **In**, the amount you get depends on a random lottery.

The lottery can produce **Yellow** or **Green**. If the lottery produces **Yellow**, you and your counterpart get 75 SEK each. If the lottery produces **Green**, you get 40 SEK and your counterpart gets 110 SEK.


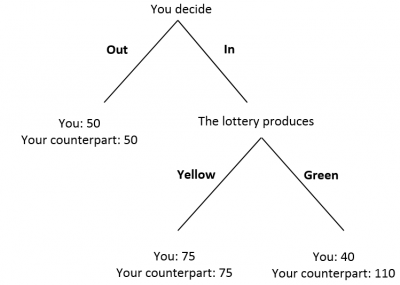


**Your task**

Think of the lottery as an urn that contains a total of 20 colored balls, each of which may be either yellow or green. If one yellow ball is drawn, the lottery produces **Yellow**. If one green ball is drawn, the lottery produces **Green**. The number of yellow and green balls has been pre-determined before the experiment.

Your task is to consider all possible values of the number of yellow balls in the urn. In other words, your task is to decide between **In** and **Out** *for all possible values of the number of yellow balls in the urn*. That is: Do you choose **In** or **Out** if 20 of 20 balls are yellow? Do you choose **In** or **Out** if 19 of 20 balls are yellow? Do you choose **In** or **Out** if 18 of 20 balls are yellow? And so on.

You will make your decisions by filling in a table similar to the one below:


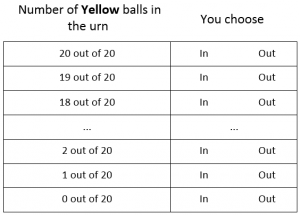


Select either In or Out for each row of the table.

**How payment is calculated**

At the end of the experiment, we will count the number of yellow balls in the urn. This will indicate which row of the table we will look at to determine your (and your counterpart's) payment.

**EXAMPLE 1:** Suppose that 19 of 20 balls are yellow. We will then select the row 2 of the table you completed. Suppose further that you chose **In** in that row. Then, **In** would be the decision of yours relevant to your earnings. At that point, there would be two possible cases: either we draw one yellow ball from the urn or we draw the green one. In the former case, you and your counterpart get 75 SEK each. In the latter case, you get 40 SEK and your counterpart gets 110 SEK.

**EXAMPLE 2:** Suppose that 1 of 20 balls are yellow and that you chose **Out** in the corresponding row. Then, **Out** will be the decision of yours that is relevant for your earnings. In this case, you and your counterpart get 50 SEK each, regardless of whether we draw a yellow or a green ball from the urn.

You will be paid in cash right after you finish the experiment. Your counterpart will be contacted via e-mail and will be asked to collect his/her payment. We will not reveal your identity to your counterpart or your counterpart's identity to you.

Remember: all the decisions you take are potentially important because you don’t yet know the actual number of yellow balls in the urn.

Click on the arrow to begin.

[Page break]


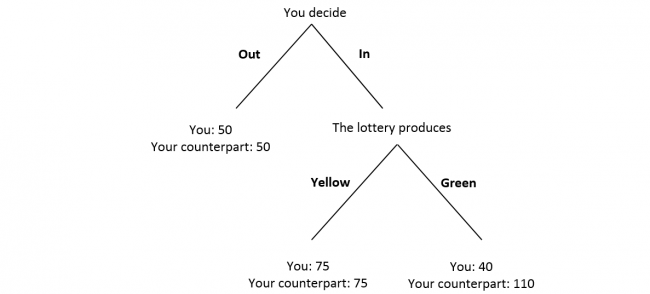


Number of **Yellow** balls in the urn:                                       You choose:

|  | In | Out |
| --- | --- | --- |
| 20 out of 20 |  |  |
| 19 out of 20 |  |  |
| 18 out of 20 |  |  |
| 17 out of 20 |  |  |
| 16 out of 20 |  |  |
| 15 out of 20 |  |  |
| 14 out of 20 |  |  |
| 13 out of 20 |  |  |
| 12 out of 20 |  |  |
| 11 out of 20 |  |  |
| 10 out of 20 |  |  |
| 9 out of 20 |  |  |
| 8 out of 20 |  |  |
| 7 out of 20 |  |  |
| 6 out of 20 |  |  |
| 5 out of 20 |  |  |
| 4 out of 20 |  |  |
| 3 out of 20 |  |  |
| 2 out of 20 |  |  |
| 1 out of 20 |  |  |
| 0 out of 20 |  |  |

[Page break]

**Instructions for the Dictator Game**

**INSTRUCTIONS TASK 3**

In this task, you will be asked how you want to distribute a sum of money between yourself and a charitable organization.

Click on the arrow to begin.

[Page break]

How much of 100 SEK do you want to give to yourself and how much do you want to give to UNICEF?

______ To me:

______ To UNICEF:

[Page break]

Please let the experimenter know you have finished task 3.

[Page break]

[Experimenter starts BART in Inquisit]

**Instructions for the Balloon Analog Risk Task (BART)**

**INSTRUCTIONS TASK 4**

You will now be presented with 30 balloons, one after the other, on the screen. For each balloon, you can click on the button that pumps up the balloon. Each time you click on the pump button, the balloon is pumped up some more.

BUT remember, balloons pop if you pump them up too much. It is up to you to decide how much you pump up each balloon. Some of these balloons may pop after just one pump. Others may not pop until they fill the whole screen.

You will receive MONEY each time you pump up the balloon. Each pump earns you 0.10 SEK. But if a balloon pops you lose the money that you earned on that balloon. To keep the money from a balloon, stop pumping before it pops and click the button marked “collect $$$”.

After each time you collect money or pop a balloon, a new balloon will be presented.

Continue to show a summary.

[Page break]

Summary

* You earn 0.10 SEK for each pump.

* You collect the money from a balloon when you click ”collect $$$”.

* You lose money from a balloon when it pops.

* There is only 30 balloons.

Begin when you are ready.

**Instructions for Self-Report Measures**

We will now ask some questions about yourself and about the experiment you just completed.

[Page break]

How pleasant was the touch?

Very unpleasant Very pleasant


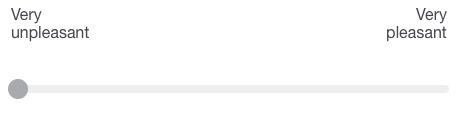


[Page break]

How relaxing was the touch?

Not at all Very

relaxing relaxing


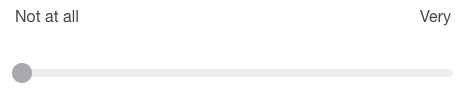


[Page break]

We will now test your understanding of the first task that you completed.

This was the basic structure of the game:


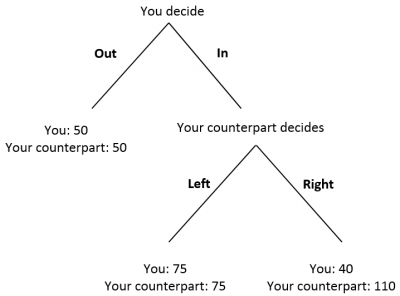


In your opinion, how difficult were the instructions to understand?


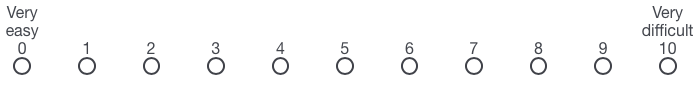


Suppose you were a participant and you filled in the table in the following way:


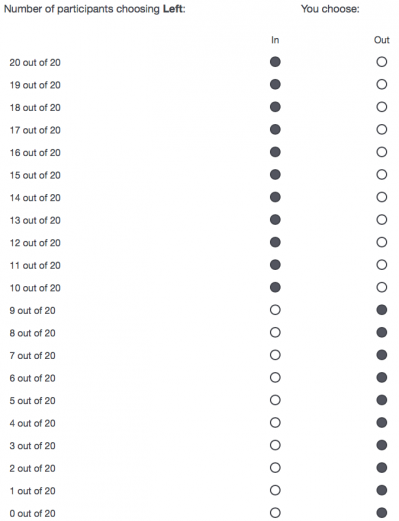


Suppose the experimenter then revealed that 14 of the previous participants chose **Left**.

a. Would your payment depend on your counterpart's choice?

- Yes
- No

Suppose your counterpart chose **Left**:

b. How much money would you get? _______

c. How much money would your counterpart get? _______

[Page break]


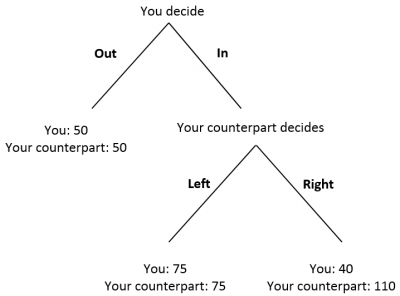


Now instead suppose you filled in the table in the following way:


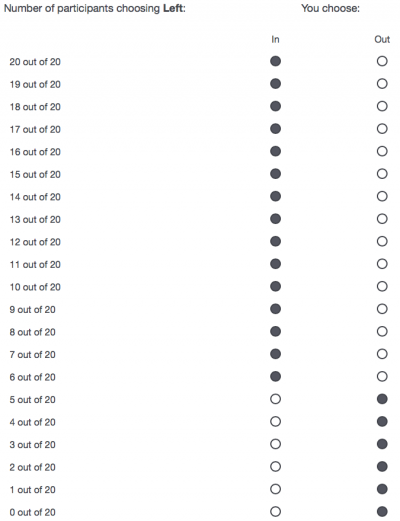


For this case suppose that the experimenter then revealed that 4 of the previous participants chose **Left**.

a. Would your payment depend on your counterpart's choice?

- Yes
- No

Suppose your counterpart chose **Right**:

b. How much money would you get? _______

c. How much money would your counterpart get? _______

[Page break]

The following questions concern how you **generally**feel in i**mportant close relationships in your life**. Think about your past and present relationships with people who have been especially important to you, such as family members, romantic partners, and close friends. Respond to each statement in terms of how you generally feel in these relationships.

|  | Not at all  characteristic  of me |  |  |  | Very  characteristic  of me |
| --- | --- | --- | --- | --- | --- |
|  | 1 | 2 | 3 | 4 | 5 |
| I find it relatively easy to get close to people. |  |  |  |  |  |
| I find it difficult to allow myself to depend on others. |  |  |  |  |  |
| I often worry that other people don't really love me. |  |  |  |  |  |
| I find that others are reluctant to get as close as I would like. |  |  |  |  |  |
| I am comfortable depending on others. |  |  |  |  |  |
| I don’t worry about people getting too close to me. |  |  |  |  |  |
| I find that people are never there when you need them. |  |  |  |  |  |
| I am somewhat uncomfortable being close to others. |  |  |  |  |  |
| I often worry that other people won’t want to stay with me. |  |  |  |  |  |
| When I show my feelings for others, I'm afraid they will not feel the same about me. |  |  |  |  |  |
| I often wonder whether other people really care about me. |  |  |  |  |  |
| I am comfortable developing close relationships with others. |  |  |  |  |  |
| I am uncomfortable when anyone gets too emotionally close to me. |  |  |  |  |  |
| I know that people will be there when I need them. |  |  |  |  |  |
| I want to get close to people, but I worry about being hurt. |  |  |  |  |  |
| I find it difficult to trust others completely. |  |  |  |  |  |
| People often want me to be emotionally closer than I feel comfortable being. |  |  |  |  |  |
| I am not sure that I can always depend on people to be there when I need them. |  |  |  |  |  |

[Page break]

What do you think was the purpose of this study? What was/were the hypothesis/-es?

[Page break]

Do you think the 20 previous participants really completed the first task before you and will be paid if that task is selected for payment, or do you think we made them up?

- They played the game and will be paid
- They played the game but will not be paid
- There were no 20 previous participants
- Other, please explain: _______

[Page break]

What is your age (in years)? _______

What is your gender?

- Male
- Female
